# Dysregulated Intraocular Pressure in Acanthamoeba Keratitis: Clinical Associations, Therapy, and Prognosis

**DOI:** 10.3390/microorganisms14061294

**Published:** 2026-06-08

**Authors:** Yaser Abu Dail, Elias Flockerzi, Mathias Roth, Gerd Geerling, Robert Bock, Thomas Reinhard, Daniel Böhringer, Philip Maier, Nóra Szentmáry, Cristian Munteanu, Berthold Seitz, Loay Daas

**Affiliations:** 1 Department of Ophthalmology, Saarland University Medical Center, 66424 Homburg, Germany; 2 Department of Ophthalmology, University Hospital Düsseldorf, 40225 Düsseldorf, Germany; 3 Eye Center at Medical Center, University of Freiburg, 79098 Freiburg, Germany; 4 Department of Ophthalmology, Semmelweis University, 1085 Budapest, Hungary; nszentmary@gmail.com; 5 The Institute for Experimental Ophthalmology, Saarland University, 66421 Homburg, Germany

**Keywords:** acanthamoeba keratitis, intraocular pressure, glaucoma, penetrating keratoplasty, corneal graft survival rate

## Abstract

This retrospective multicenter cohort study investigated the incidence, treatment, and prognostic impact of dysregulated intraocular pressure (DIOP; >24 mmHg requiring therapy) in patients with acanthamoeba keratitis (AK). Eighty-one eyes from 81 patients with confirmed AK were included; co-infections were excluded. DIOP and best corrected visual acuity (BCVA) were assessed at baseline, follow-up, and after penetrating keratoplasty (PKP). Overall, 28% of eyes were managed conservatively, 46% required one PKP, and 26% required two. DIOP occurred in 4% before AK, 11% after AK onset, and 10% after the first PKP. Eyes with DIOP prior to PKP had significantly worse final BCVA compared to those without DIOP (0.5 ± 0.7 vs. 1.8 ± 1.1 logMAR, *p* < 0.001) and required PKP more frequently (100% vs. 67%, *p* = 0.01). Time to correct diagnosis was significantly longer in the AK-associated DIOP group (64 ± 50 vs. 50 ± 68 days, *p* = 0.03). DIOP was also associated with higher rates of prior cortisone use and complications, including fixed dilated pupil, cataract, anterior synechiae, and anterior chamber irritation. Overall, DIOP represents a major complication in AK and is linked to poorer visual outcomes. Earlier therapy may help reduce DIOP and hence improve prognosis.

## 1. Introduction

Acanthamoeba keratitis (AK) is an infrequent and occasionally sight-threatening infectious keratitis (IK) caused by Acanthamoeba species [1]. The first AK case was described by Naginton et al. following an ocular trauma [2]. Acanthamoeba is the cause in 0–5% of all IK [3]. Incidence of AK is thought to range from 0.33 to 1.49 cases per 10,000 contact lens (CL) wearers and 0.13–2.7 per million per year in the general population [4,5,6]. Ameobae of the genus *Acanthamoeba* can also cause other human infections, including granulomatous amebic encephalitis as well as infections of the lungs and skin [7]. Early therapy of AK is crucial for a good prognosis [8,9,10], as patients with long-standing AK may develop severe complications such as persistent epithelial defects (PEDs), corneal neovascularization, and limbal stem cell deficiency. The infection could also extend to the limbus or to the sclera, leading to the loss of vision or even of the eye [1,11,12,13,14,15]. Another important complication in AK patients is dysregulated intraocular pressure (DIOP). The incidence of Acanthamoeba-associated glaucoma (AAG) in eyes with AK is reported to be between 6.2 and 33% [16,17,18]. Several factors have been shown to be associated with DIOP in AK patients, including topical steroid use [18]. Mature cataract and fixed dilated pupil were also reported to be frequent in AK patients [17]. The rate of secondary glaucoma after a therapeutic penetrating keratoplasty (TK) is also estimated to be as high as 36% [17,19,20,21].

The prognosis of AK-associated DIOP is controversial in the literature. While some studies show that DIOP is associated with a significantly worse prognosis [16,17], a study from Al Owaifeer et al. showed that DIOP had no significant effect on the prognosis [18]. Additionally, another study from our group showed that the rate of secondary glaucoma after low-load keratoplasty (LLKP; a penetrating keratoplasty (PKP) performed after intensive anti-amoebic therapy (AAT) for several weeks, and before discontinuing AAT, in eyes with deep stromal infiltration progressing to limbus and/or visually significant corneal scarring) was only 14% [14], indicating that the timing and preoperative findings in AK patients can influence the development rate of DIOP after keratoplasty.

The aim of this study was to report the incidence of AK-associated DIOP, defined as DIOP developing or worsening after the onset of AK, and its association with clinical findings and treatment outcomes, including conservative management and surgical interventions such as corticosteroid eye drops, PKP, and repeat PKP, through direct comparison between patients with and without AK-associated DIOP in a multicenter cohort of 81 patients.

## 2. Patients and Methods

In this retrospective study, patients who were treated for AK in Homburg, Freiburg, or Düsseldorf University Hospitals were included. All patients were treated between 1993 and 2024 and had adequate documentation of medical history and clinical findings. DIOP was defined as an intraocular pressure > 24 mmHg with the need for pressure-lowering therapy [22]. AK-associated DIOP was defined as DIOP developing or worsening after the onset of AK. The study was conducted in accordance with the Declaration of Helsinki and was approved by the Ethics Committee of the Medical Association of Saarland, Germany (Nr.84/17). Written informed consent was waived due to the retrospective nature of the study. All data were collected as part of the regular clinical examination and treatment process.

AK diagnosis was confirmed by PCR, histology, microbiology, and/or in vivo confocal microscopy (IVCM). Exclusion criteria were missing data as well as mixed corneal infection, because these could confound the results. During the above-mentioned period, 182 eyes were treated, of which 69 were excluded because of missing data and 32 because of mixed infection. A total of 81 eyes from 81 patients were included in the current study.

Demographic data, best corrected visual acuity (BCVA) (logMAR), presence of DIOP, the time point of DIOP diagnosis (before AK, after AK before PKP, after PKP), presence of posterior synechiae, anterior synechiae, anterior chamber irritation (cells/hypopyon/fibrin), cataract development before PKP, presence of fixed dilated pupil, date of correct diagnosis, defined as the date of initiating the correct topical AK therapy (polyhexamethylene biguanide (PHMB) or chlorhexidine, and propamidine isethionate), use of local cortisone therapy before correct diagnosis, performing first and second PKP, period from begin of symptoms to correct diagnosis in days, presence of DIOP after the first and second PKP, development of hypotony, and BCVA at last visit were documented. The number of topical IOP-lowering eye drops and surgical therapy for glaucoma has also been documented.

BCVA was presented in logMAR units to allow for comparison with the results of other studies. The logMAR equivalent for counting fingers was considered 1.8, for hand motion 2.3, for light perception 2.7, and for no light perception 3.0 [23,24].

The total cohort was first divided into two subgroups according to the development of DIOP **before PKP** for AK. The first group (“no DIOP before or without PKP”) included patients who did not develop DIOP prior to undergoing PKP, as well as those who neither developed DIOP nor required PKP during the entire follow-up period. The second group (“with DIOP before or without PKP”) comprised patients who developed DIOP before undergoing PKP during the follow-up period, as well as those who developed DIOP without requiring PKP during the follow-up period.

Another sub-analysis was then done using two groups with and without AK-associated DIOP, regardless of whether DIOP developed before or after PKP.

In addition, the Kaplan–Meier survival rate of the first PKP, with “survival” defined as not requiring a second PKP during the entire follow-up, was calculated.

For statistical analysis, Microsoft Excel version 2402 (Microsoft Corp., Redmond, WA, USA) was used. Continuous parameters were reported as the mean and standard deviation. Categorical parameters were reported as percentages. Continuous variables with a normal distribution were compared using Student’s *t*-test. Continuous variables which were not normally distributed were compared using the Mann–Whitney U test. Categorical parameters were compared using the Chi-Square test. Graft survival was assessed using the log-rank test. A *p*-value of less than 0.05 was considered statistically significant.

## 3. Results

A total of 81 eyes of 81 patients were included in this study. At baseline, 12 patients (15%) had DIOP before or without PKP (group A) (age: 39 ± 17 [30–61] years); three of them had glaucoma before AK which worsened after the infection. A total of 69 eyes had no DIOP before or without PKP (group B) (age: 47 ± 11 [12–80] years). Demographic data as well as functional and surgical outcomes of both groups are summarized in Table 1. The average time from the beginning of symptoms to correct diagnosis was 49 ± 64 [0–415] days in group B and was significantly shorter compared to 76 ± 58 [18–217] days in group A at baseline (*p* = 0.04). BCVA improved significantly only in group B from baseline to last visit (1.3 ± 0.8 [0–2.7] logMAR to 0.5 ± 0.7 [0–3] logMAR, *p* < 0.001). BCVA showed no improvement in group A (1.8 ± 0.8 [0.5–2.7] to 1.8 ± 1.1 [0.2–3], *p* = 1). The difference in BCVA at baseline between the two groups was not significant (*p* = 0.08) but became significant at the last visit (*p* = 0.002). Use of topical cortisone therapy was significantly higher in group A compared to group B (80% vs. 42%, *p* = 0.02). Patients in group A required PKP more frequently than those in group B (100% vs. 67%, *p* = 0.01). However, the number of second PKPs performed did not differ significantly between the two groups (50% vs. 34%, *p* = 0.3).

**Figure 1:**
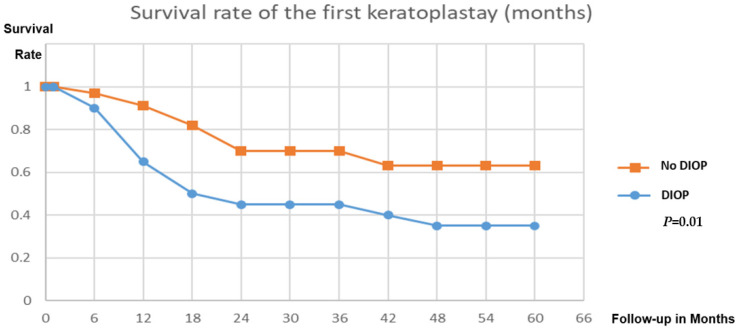
**Kaplan–Meier survival rate of the first corneal graft.** Survival rate of the keratoplasty, defined as not requiring a second keratoplasty during the entire follow-up, in eyes with and without Acanthamoeba keratitis-associated dysregulated intraocular pressure (DIOP) (log-rank test *p* = 0.01).

Overall, 20 patients had an AK-associated DIOP (before and/or after PKP) (group 1) (25%) compared to 61 patients who had no AK-associated DIOP (group 2) (age: 38 ± 17 [26–80] and 46 ± 14 [12–75] years, respectively, *p* = 0.06). Demographic data as well as functional and surgical outcomes of both groups are summarized in Table 2. BCVA improved significantly only in group 2 from baseline to last visit from (1.2 ± 0.8 [0–2.7] logMAR to 0.4 ± 0.6 [0–2.7] logMAR, *p* < 0.001). BCVA showed no improvement in group 1 (1.8 ± 0.7 [0.5–2.7] to 1.6 ± 1.0 [0.2–3], *p* = 0.4). The difference in BCVA at baseline between the two groups was significant (*p* = 0.008) and remained significant at last visit (*p* < 0.001). Hypotony developed in four patients in group 1 compared to two patients in group 2 (20% vs. 2%, *p* = 0.01). Anterior synechiae, anterior chamber irritation, cataract development before PKP, and fixed dilated pupil were all significantly higher in group 1 compared to group 2 (*p* < 0.007). The rate of the first PKP and the second PKP was higher in group 1 compared to group 2 (first PKP: 100% vs. 62%, *p* = 0.001, second PKP: 55% vs. 26%, *p* = 0.03, log-rank test *p* = 0.01) (Figure 1). The time period from the beginning of symptoms to correct diagnosis was also significantly longer in group 1 compared to group 2 (64 ± 50 [0–415] vs. 50 ± 68 [18–217] days, *p* = 0.03). On the other hand, while the time from the initiation of anti-amoebic therapy to the first PKP was longer in group 1 compared to group 2, the difference was not statistically significant (385 ± 559 [0–1778] vs. 110 ± 131 [3–460] days, *p* = 0.4).

The Kaplan–Meier survival rate of the first graft at four years was significantly higher in group 2 (63%) than in group 1 (35%) (*p* = 0.01).

Supplementary Table S1 summarizes the conservative and surgical glaucoma therapies of the 20 patients with AK-associated DIOP.

## 4. Discussion

Glaucoma is a well-described complication of AK [17]. The etiology of AK-associated glaucoma is likely multifactorial. The use of corticosteroid therapy prior to correct diagnosis was shown to be significantly more frequent in patients developing AK-associated glaucoma, both in the study of Al Owaifeer et al. and Hussain et al. [17,18] as well as in the current study. This could possibly occur as a side effect of the medication itself or due to the delay in the diagnosis under cortisone therapy, leading to a more severe form of AK with consequent complications such as glaucoma [25]. Another possible mechanism is the (partial) closure of the anterior chamber angle through anterior synechiae, mature cataract, anterior chamber irritation, and fixed dilated pupil. The frequency of these unfavorable findings was, indeed, significantly higher in patients with AK-associated DIOP in the current study. Similarly, Hussain et al. [17] reported a high rate for mature cataract and/or fixed dilated pupil in patients with glaucoma (65%). In addition, histologic examination in the study of Kelley et al. showed a chronic inflammation of the trabecular meshwork and angle closure and no evidence for pathogen infection of the angle structures [16]. The chronic inflammation associated with AK might also influence structural changes in the trabecular meshwork through inhibiting cell adhesion and proteasome activity in trabecular meshwork cells, while promoting premature senescence and extracellular matrix crosslinking. This could result in extracellular matrix accumulation and increase aqueous humor outflow resistance in glaucoma [26]. To the best of our knowledge, the current study is the first to show a significantly higher frequency of anterior chamber irritation and anterior synechiae in patients who develop AK-associated DIOP. Further studies are warranted to explore this association and determine the pathophysiology of DIOP in AK patients.

The prevalence of DIOP after AK is estimated in the literature between 6.2 and 33% [16,17,18]. The prevalence in our study (25%) was within this range. The variability in estimation between studies is likely due to differences in criteria for defining glaucoma or OHT (e.g., IOP of 21 mmHg in the study from Hussain et al. [17] vs. 24 mmHg in our study), different inclusion and exclusion criteria, and possibly differences in the severity of AK cases treated in study centers.

The functional and morphological prognosis in AK-associated DIOP patients was also limited compared to that of those without DIOP. The BCVA in patients with AK-associated DIOP was significantly worse at baseline and showed no significant improvement at the last visit. This is in accordance with the findings of Kelley et al., in which four of six patients with glaucoma had BCVA of light perception or worse at last visit, and of those from Hussain et al., showing that only 21% of eyes with AK-associated DIOP had a BCVA < 1 logMAR. Similarly, glaucoma was responsible for 33% of eyes with poor visual outcomes in a case series published by Bacon et al. [27]. However, it is important to note that although DIOP is associated with a limited visual prognosis, it may not always be the direct cause of it. These eyes also frequently suffer from significant corneal opacities and surface irregularity, both of which may additionally contribute to poor visual outcomes.

Additionally, a PKP was more frequently performed in patients with AK-associated DIOP compared to those without. The overall percentage of PKP was also high in the current cohort. This is likely due to the higher percentage of complicated cases being treated in our three tertiary participating centers. As patients with DIOP showed a significantly higher percentage of several other complications like fixed dilated pupil and anterior chamber irritation, it is difficult to determine the causality between DIOP and the first PKP. Further studies with better statistical power and multiple regression analysis may be required to further explore this relation.

The rate of a second PKP was also higher in patients with AK-associated DIOP. Similar findings were published by Kashiwabushi et al., who reported a worse survival rate of corneal graft in patients with AK and glaucoma compared to those without glaucoma [20]. Even in the absence of AK, Reinhard et al. showed that glaucoma is associated with a worse graft survival rate after PKP [28].

One interesting finding in the current study is that the period from the beginning of symptoms to correct diagnosis was significantly longer in patients with AK-associated DIOP. This is possibly due to the role of the early therapy in [14,15]. Additionally, a longer infection period is associated with a higher frequency of anterior uveitis and hypopyon [27], findings which were shown to be more frequent in patients with AK-associated DIOP in the current study. These findings underscore the role of a severe and prolonged course of AK in the development of DIOP. On the other hand, while the time from initiation of anti-amoebic therapy to PKP tended to be longer in patients with AK-associated DIOP, this tendency did not reach statistical significance. A similar tendency was also reported by Hussain et al., who also found that 57% of patients with AK-associated glaucoma had been treated for more than one year for AK [17]. Further studies with better statistical power are hence required to further explore this potential relation.

The treatment of AK-associated DIOP poses a significant challenge. A total of 10% of the eyes with AK-associated DIOP were eventually enucleated. The same rate was reported by Hussain et al. [17]. A total of 75% of the eyes required topical IOP-lowering eye drops at the last visit, and 30% of them required surgical treatment for glaucoma after PKP. Surgical glaucoma therapy was also performed in 60% of the patients in the study by Hussain et al., which underscores the high therapeutic burden in these patients.

This study is limited due to its retrospective design and limited cohort size. Another inherent limitation of the study is the frequent presence of corneal edema and corneal surface irregularities in AK patients, which may lead to less accurate measurement of the intraocular pressure. Nevertheless, in all patients that we included in the DIOP group, the intraocular pressure measurements were considered to be sufficiently reliable to justify initiation of anti-glaucoma treatment by the treating specialists. Further studies with better statistical power and/or meta-analyses are warranted to explore the causality between DIOP and the various concurrent clinical findings, as well as the best therapeutic approach in patients with AK-associated DIOP.

In summary, AK-associated DIOP is a frequent complication of AK, which is associated with a higher percentage of several clinical findings, such as mature cataract, anterior chamber irritation, anterior synechiae, and fixed dilated pupil, a worse BCVA outcome, and a higher percentage for PKP and repeat PKP, as well as a major need for conservative and surgical glaucoma therapy. Avoiding the use of topical cortisone before making the correct diagnosis and early adequate anti-amoebic therapy might help reduce DIOP and hence improve the prognosis in AK patients.

## Figures and Tables

**Table 1:** Demographic characteristics and surgical and functional outcomes of patients with Acanthamoeba keratitis with and without dysregulated intraocular pressure (DIOP) before or without PKP^a^ at baseline visit.

	No DIOP Before or Without PKP ^a^ (69 Eyes)	DIOP Before or Without PKP ^a^ (12 Eyes)	*p*-Value
Age ^b^ (yrs): Mean ± SD ^a^	47 ± 11 [12–80]	39 ± 17 [30–61]	0.12 ^c^
Gender (Male: Female)	29:40	4:8	0.6 ^d^
BCVA **^a^** at baseline (logMAR) Mean ± SD ^a^	1.3 ± 0.8 [0–2.7]	1.8 ± 0.8 [0.5–2.7]	0.08 ^c^
BCVA at last visit (logMAR) Mean ± SD ^a^	0.5 ± 0.7 [0–3]	1.8 ± 1.1 [0.2–3]	0.002 ^c^
*p*-Value	<0.001 ^e^	1 ^e^	
Local cortisone therapy before correct diagnosis	25/60 (42%)	8/10 (80%)	0.02 ^d^
First PKP performed	46 (67%)	12 (100%)	0.01 ^d^
Time from begin of symptoms to correct diagnosis (Days)	49 ± 64 [0–415]	76 ± 58 [18–217]	0.04 ^f^
Second PKP performed	15/46 (34%)	6/12 (50%)	0.3 ^d^

^a^ PKP: penetrating keratoplasty, SD: standard deviation, BCVA: best corrected visual acuity, “no DIOP before or without PKP”: This group included patients who did not develop DIOP prior to undergoing PKP, as well as those who neither developed DIOP nor required PKP during the entire follow-up period, “with DIOP before or without PKP”): this group comprised patients who developed DIOP before undergoing PKP during the follow-up period, as well as those who developed DIOP without requiring PKP during the entire follow-up period, ^b^ Age calculated at date of first presentation in the tertiary clinic, ^c^ Comparison between glaucoma before or without PKP group and no glaucoma before or without PKP group at baseline group using Student’s *t*-test, ^d^ Comparison between glaucoma before PKP group and no glaucoma before or without PKP group using Chi-square test, ^e^ Comparison between BCVA at baseline and at last visit in groups with and without glaucoma before or without PKP at baseline using paired *t*-test. ^f^ Comparison between glaucoma before or without PKP group and no glaucoma before or without PKP group using Mann–Whitney U test.

**Table 2:** Surgical and functional outcomes of patients with acanthamoeba keratitis (AK) with and without dysregulated intraocular pressure (DIOP) during the entire course of infection (AK-associated DIOP).

	No AK-Associated DIOP (61 Eyes)	AK-Associated DIOP (20 Eyes)	*p*-Value
BCVA **^a^** at baseline (logMAR) Mean ± SD ^a^	1.2 ± 0.8 [0–2.7]	1.8 ± 0.7 [0.5–2.7]	0.008 ^b^
BCVA at last visit (logMAR) Mean ± SD ^a^	0.4 ± 0.6 [0–2.7]	1.6 ± 1.0 [0.2–3]	<0.001 ^b^
*p*-Value	<0.001 ^d^	0.4 ^d^	
Hypotony	2 (3%)	4 (20%)	0.01 ^c^
Posterior synechiae	5/56 (9%)	4/17 (24%)	0.1 ^c^
Anterior synechiae	2/55 (4%)	6/18 (33%)	<0.001 ^c^
Anterior chamber irritation (cells/hypopyon/fibrin)	12/55 (22%)	7/16 (44%)	0.004 ^c^
Cataract development before PKP ^a^	5/58 (9%)	6/17 (35%)	0.006 ^c^
Fixed dilated pupil	3/61 (5%)	10/20 (50%)	<0.001 ^c^
Time from beginning of symptoms to correct diagnosis (days)	50 ± 68 [18–217]	64 ± 50 [0–415]	0.03 ^e^
Time from initiation of AAT ^a^ to first PKP (days)	110 ± 131 [3–460]	385 ± 559 [0–1778]	0.4 ^e^
First PKP performed	38 (62%)	20 (100%)	0.001 ^c^
Second PKP performed	10/38 (26%)	11/20 (55%)	0.03 ^c^

^a^ PKP: penetrating keratoplasty, SD: standard deviation, BCVA: best corrected visual acuity, AAT: anti-amoebic therapy, ^b^ Comparison between AK-associated DIOP group and no AK-associated DIOP group at baseline group using Student’s *t*-test, ^c^ Comparison between AK-associated DIOP group and no AK-associated DIOP group using Chi-square test, ^d^ Comparison between BCVA at baseline and at last visit in groups with and without AK-associated DIOP at baseline using paired *t*-test. ^e^ Comparison between AK-associated DIOP group and no AK-associated DIOP group using Mann–Whitney U test.

## Data Availability

The data supporting the conclusions of this article will be made available by the authors on reasonable request.

## Supplementary Materials

The following supporting information can be downloaded at: https://www.mdpi.com/article/10.3390/microorganisms14061294/s1, Supplementary Table S1: Development of glaucoma therapy in patients with dysregulated intraocular pressure associated with Acanthamoeba keratitis.

## Abbreviations

AK	Acanthamoeba keratitis
DIOP	Dysregulated intraocular pressure
BCVA	Best corrected visual acuity
PKP	Penetrating keratoplasty
IK	Infectious keratitis
CL	Contact lens
PED	Persistent epithelial defect
AAG	Acanthamoeba-associated glaucoma
LLKP	Low load keratoplasty
AAT	Anti-amoebic therapy
IVCM	In vivo confocal microscopy
PHMB	Polyhexamethylene biguanide
OHT	Ocular hypertension
