# Supplementary material for: Dysregulated Intraocular Pressure in Acanthamoeba Keratitis: Clinical Associations, Therapy, and Prognosis
Source: Microorganisms. 2026 Jun 8;14(6):1294. doi: 10.3390/microorganisms14061294 (PMC13303941; doi:10.3390/microorganisms14061294)
Supplement: Supplementary file 1 [file microorganisms-14-01294-s001.zip › microorganisms-4281971-supplementary.pdf]

Supplementary Table S1. Development of glaucoma therapy in patients with dysregulated intraocular pressure associated with Acanthamoeba keratitis.

	Glaucoma before AK[a]	Number of IOD[a] reducing eye dops before AK	Type and number of glaucoma operations before AK	Glaucoma after AK before PKP[a]	Number of IOD reducing eye dops after AK before PKP	Number of glaucoma operations after AK before PKP	Glaucoma after PKP	Number of IOD reducing eye drops after PKP	Type and number of glaucoma operations after PKP	Important information
Patient 1	Yes	4	CPC[a]	Yes	4	0	Yes	2	0	Enucleation
Patient 2	Yes	1	0	Yes	2	0	Yes	4	0	BCVA[a] <1
Patient 3	Yes	1	0	Yes	1	0	Yes	0	Trabeculectomy	BCVA >1 logMAR
Patient 4	No	0	0	Yes	1	0	Yes	0	0	BCVA <1
Patient 5	No	0	0	Yes	3	0	Yes	3	0	Hypotony/ Enucleation
Patient 6	No	0	0	Yes	2	0	Yes	0	2x CPC[a]	BCVA light perception
Patient 7	No	0	0	Yes	1	0	Yes	1	0	BCVA <1
Patient 8	No	0	0	Yes	2	0	Yes	4	2x CPC	BCVA light perception
Patient 9	No	0	0	Yes	2	0	Yes	0	0	BCVA hand motion
Patient 10	No	0	0	Yes	1	0	Yes	0	0	BCVA >1 logMAR/ Hypotony
Patient 11	No	0	0	Yes	4	0	Yes	3	1x CPC	BCVA hand motion
Patient 12	No	0	0	Yes	1	0	Yes	3	1x CPC	BCVA >1 logMAR
Patient 13	No	0	0	No	0	0	Yes	1	0	BCVA >1 logMAR
Patient 14	No	0	0	No	0	0	Yes	4	0	BCVA nulla lux/ Hypotony
Patient 15	No	0	0	No	0	0	Yes	0	0	BCVA <1
Patient 16	No	0	0	No	0	0	Yes	1	0	BCVA <1
Patient 17	No	0	0	No	0	0	Yes	1	0	BCVA >1 logMAR
Patient 18	No	0	0	No	0	0	Yes	3	2x CPC	Hypotony
Patient 19	No	0	0	No	0	0	Yes	3	0	BCVA <1
Patient 20	No	0	0	No	0	0	Yes	1	0	BCVA hand motion

[a]AK: Acanthamoeba keratitis, IOP: intraocular pressure, PKP: penetrating keratoplasty, BCVA: best corrected visual acuity. CPC: Cyclophotocoagulation of the ciliary body.
